# The Impact of Smartphone Addiction on Job Performance in Medical Residents

**DOI:** 10.1192/j.eurpsy.2026.10832

**Published:** 2026-07-31

**Authors:** N. Rmadi, R. Affes, N. Kotti, F. Dhouib, M. Hajjaji, K. Jmal Hammami

**Affiliations:** 1 Occupational medicine department, Hedi Chaker university hospital; 2 Family medicine department, Faculty of medicine of sfax, Sfax, Tunisia

## Abstract

**Introduction:**

While smartphones are indispensable tools for communication and medical knowledge access, excessive use may impair occupational performance.

**Objectives:**

To evaluate the effect of smartphone addiction on overall work performance, among medical residents.

**Methods:**

A cross-sectional survey was conducted among 429 residents using the SAS-SV to measure smartphone addiction and the Individual Work Performance Questionnaire (IWPQ) to assess task performance, contextual performance, and counterproductive behaviors. Comparative analyses were performed between addicted and non-addicted residents.

**Results:**

Residents classified as addicted to smartphones (49.9%) showed significantly lower global work performance (2.88 ± 0.48 vs. 3.04 ± 0.50, p<0.001) compared with their non-addicted peers. Addiction was strongly associated with higher counterproductive behaviors (2.66 ± 0.89 vs. 2.30 ± 0.72, p<0.001). However, no significant differences were observed for task (p=0.070) or contextual performance (p=0.966).

**Conclusions:**

Smartphone addiction appears to undermine overall professional performance primarily by increasing maladaptive behaviors rather than directly affecting task or contextual performance. Residency programs should incorporate preventive strategies and awareness sessions on problematic smartphone use to maintain efficiency and quality of care.

**Disclosure of Interest:**

None Declared

